# Breast cancer risk among first-generation migrants in the Netherlands

**DOI:** 10.1038/sj.bjc.6601821

**Published:** 2004-04-20

**Authors:** O Visser, K van der Kooy, A M van Peppen, F G Öry, F E van Leeuwen

**Affiliations:** 1 Comprehensive Cancer Centre Amsterdam, PO Box 9236, 1006 AE Amsterdam, The Netherlands; 2 Comprehensive Cancer Centre West, Schipholweg 5A, 2316 XB Leiden, The Netherlands; 3 TNO-PG, PO Box 2215, 2301 CE Leiden, The Netherlands; 4 Pacemaker, Overtoom 538hs, 1054 LL Amsterdam, The Netherlands; 5 Department of Epidemiology, Netherlands Cancer Institute, Plesmanlaan 121, 1066 CX Amsterdam, The Netherlands

**Keywords:** breast cancer, incidence, migrants

## Abstract

We investigated breast cancer incidence in migrants in the Netherlands in 1988–1998. The standardised incidence ratio for breast cancer in Northwest-Netherlands was statistically significantly reduced for women born in Surinam (0.56), Turkey (0.29) and Morocco (0.22). The proportion of women with advanced stages (III and IV) did not differ significantly between migrants and women born in the Netherlands.

Breast cancer, the commonest cancer in women world-wide, has a particularly low incidence in Central- and East-Africa, as well as in East-Asia (Ferlay *et al*, 1998). In contrast, most Western countries have a high incidence, while the Netherlands is among the highest in the world (Van der Sanden *et al*, 1995). Differences in breast cancer incidence have been observed in the United States among different ethnic groups (Ziegler *et al*, 1993; Brinton *et al*, 1997). Studies of Japanese migrants to Hawaii have shown that breast cancer incidence adjusts to the incidence in the new homeland within one or two generations pointing to life style and environmental factors in aetiology (McPherson *et al*, 2000).

In recent decades, immigration has changed the composition of the population of the Netherlands, especially in the large cities, so that in 2002, 10% of its population and even 28% of the population of Amsterdam was foreign-born (StatLine. Statistics Netherlands). Migrants originate primarily from the former Dutch colonies (Indonesia (former name: Dutch East-Indies), Surinam and the Netherlands Antilles), Turkey and Morocco.

We have investigated differences in breast cancer incidence between migrants and women born in the Netherlands.

## MATERIALS AND METHODS

### Cancer registry data

Our data have come from the cancer registries of the Comprehensive Cancer Centre Amsterdam (CCCA) and the Comprehensive Cancer Centre West (CCCW), both part of the nation-wide Netherlands Cancer Registry (Van der Sanden *et al*, 1995). Information is extracted by registration clerks from medical records in all regional hospitals. If available in the hospital, the country of birth is routinely collected. Because the country of birth is unknown in many hospitals, only cases with residence in The Hague and the province of North-Holland have been included in this study. For women who participated in the breast cancer screening and who had agreed to record linkage of screening data to the cancer registry, the country of birth in the cancer registry has been validated with the country of birth as available in the screening data. In case of discrepancy or missing data in the cancer registry, the country of birth as available in the screening data has been used.

### Population data according to country of birth.

Statistics Netherlands receives population data from municipal population registers via the computerised population network that operates since October 1994. Annual overviews (as of 1995) of the population according to age group, sex and country of birth (Turkey, Morocco, Surinam, the Netherlands Antilles and Indonesia) for North-Holland and the city of The Hague have been used. For 1988–1994, estimations of Statistics Netherlands for 1992 and 1989–1994 (Surinam and the Netherlands Antilles only) have been used, with extrapolations for the remaining years. North-Holland and The Hague combined represent 18% of the total population of the country, but approximately one-third of the migrant population of the Netherlands.

### Statistical methods

Using the population data by 5-year age group and country of birth and age-specific breast cancer incidence rates from the Netherlands Cancer Registry for 1989–1998, expected numbers of breast cancer (E) for North-Holland and The Hague were calculated for women born in Turkey, Morocco, Surinam, the Netherlands Antilles and Indonesia, as well as for the total female population. The observed numbers (O) of breast cancer were adjusted for cases with unknown country of birth, assuming that the distribution according to country of birth for unknown cases was equal to the distribution of cases with known country of birth. Standardised incidence ratios (SIRs) were calculated as the ratio between the adjusted observed and expected numbers. Exact 95% confidence intervals (CI) based on the Poisson distribution of the adjusted O were calculated using STATA 7.0 (StataCorp. Stata Statistical Software: Release 7.0. College Station, TX: Stata Corporation).

## RESULTS

### Breast cancer incidence

In 1988–1998, a total of 16 499 invasive breast cancers were diagnosed in North-Holland (28% resident in Amsterdam) and 3517 in The Hague (Table 1

**Table 1:** Number of invasive breast cancers in Northwest-Netherlands (The Hague and North-Holland) according to TNM-stage and country of birth, 1988–1998

	**All stages**		**TNM-stage at diagnosis (*n*=16 297)^a^**	
**Country of birth**	**Number**	**%^b^**	**Stages I–II (%)**	**Stages III–IV (%^c^)**
Netherlands	16 702	83.3	85	15
Western countries	1233	6.2		
Indonesia	583	2.9	85	15
Germany	245	1.2	83	17
Other Western countries	405	2.0	82	18
Non-Western countries	466	2.3		
Surinam	218	1.1	83	17
Turkey	40	0.2	87	13
Morocco	26	0.1	86	14
Netherlands Antilles	59	0.3	78	22
Other non-Western countries	123	0.6	82	18
Unknown	1615	8.1	84	16
				
Total	20 016	100.0	85	15

a ^a^Unknown TNM-stage was included in I–II; cases for which TNM was not applicable (*n*=202) and cases with residence in The Hague (*n*=3517) excluded.

b Percentage calculated vertically.

c Percentage calculated horizontally.

). In total, 83% of the women were born in the Netherlands. The percentage of foreign-born was 12% in The Hague, 13% in Amsterdam and 6% in North-Holland (excluding Amsterdam). The country of birth was unknown for 8% of the cases.

Figure 1

**Figure 1:**
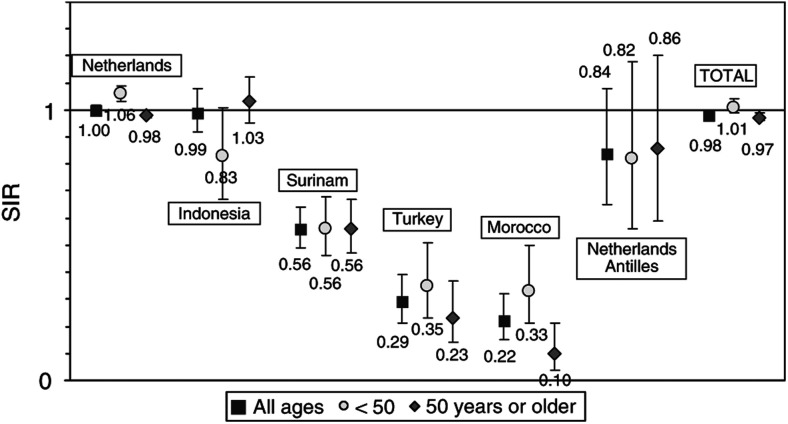
Standardised incidence ratio (SIR) for invasive breast cancer according to country of birth in 1988–1998 in North-Holland and The Hague, the Netherlands (bars represent 95% CIs; reference population: the Netherlands 1989–1998).

shows that the overall SIR in North-Holland and The Hague was similar to the national average (SIR 0.98; 95% confidence interval (CI): 0.97, 1.00), slightly higher in women below the age of 50 (SIR 1.01) than in women of 50 years or older (SIR 0.97). For women born in Indonesia the SIR was similar to the national average (SIR for all ages: 0.99, 95% CI: 0.92, 1.08), but for women below the age of 50 the rate was lower (SIR 0.83, 95% CI: 0.67, 1.01). For women born in the Netherlands Antilles, the SIR was 0.84 (95% CI: 0.65, 1.08) and for women born in Surinam a statistically significantly decreased rate of 0.56 (95% CI: 0.49, 0.64) was found. Very low rates were observed for women born in Turkey (SIR 0.29, 95% CI: 0.21, 0.39) and Morocco (SIR 0.22, 95% CI: 0.15, 0.32). In women born in Turkey or Morocco, the SIRs were lower among those 50 years or older than among younger women below the age of 50 (0.23 *vs* 0.35 and 0.10 *vs* 0.33, respectively).

### Stage distribution

Table 1shows the distribution by TNM-stage and country of birth for women with breast cancer in North-Holland. Cases of unknown stage (2% of all cases), mainly due to unknown tumour size, were considered as localised. In all, 85% of all cancers were localised (stage I or II), and 15% had locally advanced disease or distant metastases (stages III or IV). The proportion of advanced stages ranged from 13% for women born in Turkey to 22% for those born in the Netherlands Antilles. A logistic regression analysis showed that, corrected for age group, the proportion with advanced disease did not differ significantly between the foreign-born and the native-born women (15%).

## DISCUSSION

The risk of breast cancer among women resident in the Netherlands but born in Surinam, Turkey and Morocco appears to be still close to that in their country of origin. Although no national incidence data are available for these countries, estimates for the year 1990 show incidence rates that were 48, 29 and 35%, respectively, of the Netherlands rate (Parkin *et al*, 1999). In the Netherlands Antilles, the incidence was 60.9 per 100 000 women (European standardised rate) in 1987–1991, about 60% of the Netherlands rate during that period (Schakenraad *et al*, 1995). The somewhat higher breast cancer risk of women born in the Netherlands Antilles resident in the Netherlands is probably due to the selective migration of higher educated women. Until recently, further education was one of the main motives for women to migrate to the Netherlands.

The low incidence in women born in Turkey and Morocco can probably be attributed to differences in breast cancer risk factors in comparison to native women, especially in relation to reproduction. Compared to native women , women born in Turkey and Morocco have more children and have their first child at an earlier age. Because these women mainly came to the Netherlands as a partner of (male) migrant workers, the percentage of unmarried women without children is negligible (StatLine. Statistics Netherlands). Reproductive factors in the Surinam-born are intermediate between those in native women and those born in Turkey or Morocco, so their intermediate incidence is not surprising. The smallest differences in reproductive factors from native women occur in women born in the Netherlands Antilles and correspondingly their breast cancer risk is close to that in women of Dutch descent.

The higher incidence in younger (below age 50) than in older women born in Morocco or Turkey, is probably due to a change in risk factors in younger women, such as lower parity. The slightly lower risk for women below the age of 50 born in Indonesia (SIR 0.83) may reflect the fact that older women are mainly of Caucasian (Dutch) origin, while younger women, especially those born after the independence of Indonesia in 1949, are more often of ethnic Indonesian origin.

In conclusion, in Northwest-Netherlands, women born in Surinam, Turkey and Morocco have a significantly lower breast cancer risk than women born in the Netherlands. It will be of interest to examine the risk of second-generation migrants in the future.
